# Effects of voxel size and iterative reconstruction parameters on the spatial resolution of ${\;}^{99m}\text{Tc}$ SPECT/CT

**DOI:** 10.1120/jacmp.v12i4.3459

**Published:** 2011-11-15

**Authors:** S. Cheenu Kappadath

**Affiliations:** ^1^ Department of Imaging Physics The University of Texas MD Anderson Cancer Center Houston TX 77030 USA

**Keywords:** SPECT, spatial resolution, iterative reconstruction, SPECT/CT

## Abstract

The purpose of this study was to evaluate the effects of voxel size and iterative reconstruction parameters on the radial and tangential resolution for ${\;}^{99m}\text{Tc}$ SPECT as a function of radial distance from isocenter. SPECT/CT scans of eight coplanar point sources of size smaller than $1\;{\text{mm}}^{3}$ containing high concentration ${\;}^{99m}\text{Tc}$ solution were acquired on a SPECT/CT system with 5/8 inch NaI(Tl) detector and low‐energy, high‐resolution collimator. The tomographic projection images were acquired in step‐and‐shoot mode for 360 views over 360° with 250,000 counts per view, a zoom of 2.67, and an image matrix of 256×256 pixels that resulted in a $0.9\times 0.9\times 0.9\;{\text{mm}}^{3}$ SPECT voxel size over 230 mm field‐of‐view. The projection images were also rebinned to image matrices of 128 × 128 and 64 × 64 to yield SPECT voxel sizes of 1.8×1.8×1.8 and $3.6\times 3.6\times 3.6\;{\text{mm}}^{3}$, respectively. The SPECT/CT datasets were reconstructed using the vendor‐supplied iterative reconstruction software that incorporated collimator‐specific resolution recovery, CT‐based attenuation correction, and dual‐energy window‐based scatter correction using different combinations of iterations and subsets. SPECT spatial resolution was estimated as the full width at half maximum of the radial and tangential profiles through the center of each point source in reconstructed SPECT images. Both radial and tangential resolution improved with higher iterations and subsets, and with smaller voxel sizes. Both radial and tangential resolution also improved with radial distance further away from isocenter. The magnitude of variation decreased for smaller voxel sizes and for higher number of iterations and subsets. Tangential resolution was found not to be equal to the radial resolution, and the nature of the anisotropy depended on the distribution of the radionuclide and on the reconstruction parameters used. The tangential resolution converged faster than the radial resolution, with higher iterations and subsets. SPECT resolution was isotropic and independent of radial distance when reconstructed using filtered back‐projection. SPECT spatial resolution and therefore quantification of SPECT uptake via partial‐volume correction in clinical images were found to depend on the nature of activity distribution within the SPECT field‐of‐view and on the specific choice of iterative reconstruction parameters.

PACS number: 87.57.uh, 87.57.cf, 87.87.nf

## I. INTRODUCTION

Quantitative measurements of spatial resolution for single‐photon emission computed tomography (SPECT) systems for comparison against manufacturer specifications at commissioning are often performed by acquiring emission data from point or line sources in air that are then reconstructed using filtered back‐projection (FBP).^(^ ^1^ ^)^ While such test methodology may be appropriate for characterization of system performance as part of acceptance testing, the measured resolution for point sources in air does not adequately represent the system performance under clinical imaging conditions. The measurement conditions for SPECT emission data in clinical situations is complicated by scatter and attenuation due to the presence of surrounding tissue that may contain background activity. In addition, modern SPECT systems use iterative reconstruction algorithms such as ordered subsets expectation maximization (OSEM), rather than FBP, because they provide superior image quality by incorporating the physics of photon detection and corrections for scatter and attenuation.^(^ ^2^ ^)^ Evaluation of spatial resolution that accounts for the presence of scatter and photon attenuation is often done semiquantitatively using a cold‐ or hot‐rod wedge‐phantom insert as part of an image quality phantom for SPECT systems.^(^ ^3^ ^)^ The behavior or convergence of iterative reconstruction algorithms is, however, nonlinear, whereby the final resolution depends on a variety of parameters such as the local contrast, image count density, and number of iterations.^(^ ^4^ ^)^

SPECT/CT‐based internal radionuclide therapies are an emerging subfield where SPECT images are used to quantify radiopharmaceutical uptake and determine target‐tumor volumes. Finite spatial resolution is known to reduce subject contrast for small objects due to partial‐volume averaging and, as a result, affect the quantification of radiopharmaceutical uptake in emission tomography. Detailed characterization of the spatial resolution and the associated partial‐volume correction is therefore critical for quantitative SPECT images and accurate SPECT‐based internal radionuclide therapy planning.

A number of investigators have previously reported on the performance of iterative reconstruction algorithms. Some of the more pertinent publications include a simulation study of the convergence of object dependent resolution in maximum likelihood algorithms,^(^ ^5^ ^)^ the estimation of the recovery coefficient based on hot spheres using OSEM,^(^ ^6^ ^)^ and a comparison of resolution between different SPECT/CT systems using machine‐specific reconstruction algorithms.^(^ ^7^ ^)^ However, the detailed characterization of SPECT system resolution under varying imaging and OSEM reconstruction conditions across the field of view has not been performed. The objectives of this work are to characterize the behavior of the radial and tangential resolution of a SPECT/CT system under scatter and attenuation using OSEM iterative reconstruction as a function of: (i) location within the scan field of view; (ii) SPECT voxel size; and (iii) number of iterations and subsets.

## II. MATERIALS AND METHODS

SPECT/CT scans of ten coplanar point sources of size smaller than $1\;{\text{mm}}^{3}$ containing high concentration ${\;}^{99m}\text{Tc}$ solution were acquired on a Symbia T SPECT/CT system (Siemens Medical Solutions, Malvern, PA) with 5/8 inch NaI(Tl) detector and low‐energy, high‐resolution collimator. The tomographic projection images were acquired in step‐and‐shoot mode for 360 views over 360° with 250,000 counts per view, a zoom of 2.67, and an image matrix of 256 × 256 pixels that resulted in a $0.9\times 0.9\times 0.9\;{\text{mm}}^{3}$ SPECT voxel size over 230 mm field of view. The projection images were also rebinned to image matrices of 128 × 128 and 64 × 64 to yield SPECT voxel sizes of 1.8×1.8×1.8 and $3.6\times 3.6\times 3.6\;{\text{mm}}^{3}$, respectively.

The point sources were constructed by first creating small drops $\left(<\;2\;{\text{mm}}^{3}\right)$ of ${\;}^{99m}\text{Tc}$ solution using a syringe on a smooth plastic surface. Glass hematocrit capillary tubes (Fisher Scientific, Pittsburgh, PA) with inner diameters of ~1 mm were brought in contact with the ${\;}^{99m}\text{Tc}$ drops at an oblique angle such that, on contact, capillary action loaded the tube with the radioactive droplet. The capillary tubes were then sealed at both ends using an appropriate clay sealant (Critoseal, McCormick Scientific, Maryland Heights, MO). The point sources were attached to a 22 cm diameter circular foam block and positioned coplanar at radii of 2 to 11 cm from the center in 1 cm increments. The azimuthal orientation at each radial location was staggered so that each point source was sufficiently far away from those at adjacent radii to prevent an overlap in reconstructed SPECT image signal.

The cylindrical foam block was positioned in the SPECT/CT scanner such that all of the coplanar point sources were located along a single transverse plane, with the center of the foam block located at the scanner isocenter. SPECT/CT data was acquired under three different experimental configurations: (A) the point sources placed inside a 22 cm diameter cylindrical phantom (Data Spectrum Inc., Hillsborough, NC) filled with low‐concentration ${\;}^{99m}\text{Tc}$ solution such that the source‐to‐background ratio was greater than 50 (point sources in warm background); (B) the point sources suspended in air; and (C) the point sources placed inside the 22 cm diameter cylindrical phantom filled with nonradioactive water (point sources in cold background). Experiment A was designed to represent clinical imaging conditions where focal uptake in lesions is often visualized over some distributed background activity level. Experiments B and C were performed to better isolate the effects of iterative reconstruction process and compensations for scatter and attenuation on the final SPECT spatial resolution. The 22 cm diameter cylindrical phantom did not allow placement of the point source at radial distance of 11 cm. Therefore, while ten sources located 2 to 11 cm were imaged in experiment B, only nine sources located 2 to 10 cm were imaged in experiments A and C. The point source located at radial distance of 10 cm was found to be too close to the edge of the cylindrical phantom and therefore was excluded from analysis. Consequently, only eight sources located 2 to 9 cm from isocenter were used in the data analysis for all three experiments.

The SPECT/CT datasets were reconstructed using the vendor‐supplied 3D OSEM iterative reconstruction software (Flash3D, Siemens Medical Solutions) that incorporated collimator‐specific resolution recovery, CT‐based attenuation correction, and dual‐energy window‐based scatter correction.^(^ ^8^ ^)^ The 3D OSEM reconstructions were performed using different combinations of iterations (IT) and subsets (SUB): IT×SUB ɛ {1 × 18, 10 × 18, 20 × 18, 30 × 18, 30 × 36, 30 × 60, 30 × 90}. SPECT reconstructions with FBP were also performed for point sources in air (experiment B). The radial and tangential profiles through the center of each point source in reconstructed SPECT images were fit to a model of Gaussian plus a constant. SPECT spatial resolution was estimated as the full width at half maximum (FWHM) of the resulting Gaussian. Radial resolution and tangential resolution for each of the point sources in all three experiments were computed for different SPECT voxel sizes and combinations of iterations and subsets to characterize SPECT spatial resolution. The mean resolution was computed as the arithmetic mean of the radial and tangential resolutions.

## III. RESULTS

### A. SPECT resolution for point sources in warm background

**Figure 1:**
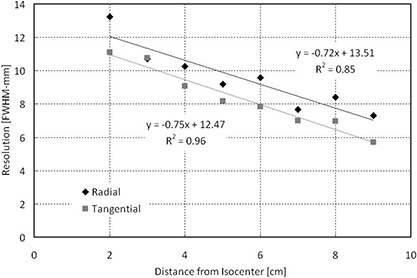
The radial and tangential resolution measured with 0.9 mm voxel size and 3D OSEM with 20 iterations and 18 subsets together with their linear fits for point sources in warm background plotted as a function of radial distance from isocenter.

The measured SPECT resolution for point sources in warm background was observed to be nonstationary (i.e., it varied as a function of spatial location within the image). The radial and tangential resolution improved (narrower FWHM) linearly with distance away from isocenter. Figure 1 shows a plot of the radial and tangential resolution as a function of distance away from isocenter for the case of 0.9 mm voxel size and 3D OSEM with 20 iterations and 18 subsets. The measured SPECT resolution was also observed to be anisotropic (i.e., the tangential resolution was not equal to the radial resolution); rather the tangential resolution was consistently better (narrower FWHM) than the radial resolution for all voxel sizes.

**Figure 2:**
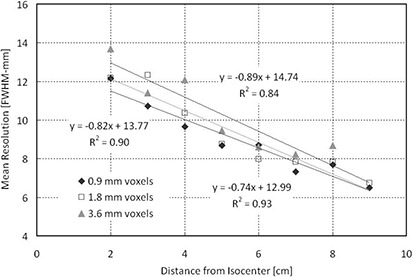
The mean resolution for point sources in warm background and their linear fits plotted as a function of radial distance from isocenter for three different SPECT voxel sizes using 3D OSEM with 20 iterations and 18 subsets.

**Figure 3:**
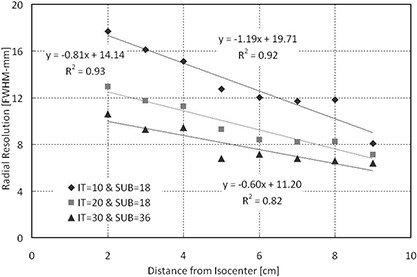
The radial resolution measured with 1.8 mm voxel size and their linear fits for point sources in warm background plotted as a function of radial distance from isocenter using three different combinations of iteration and subsets.

While a linear variation of resolution with distance away from isocenter was observed for all voxel sizes and combinations of iterations and subsets, its rate of change (slope) with radial distance depended both on the SPECT voxel size and on the product of iterations and subsets (i.e., IT×SUB). Not only did both radial and tangential resolution improve (narrower FWHM) with smaller voxel sizes, but the magnitude of change from center to periphery also decreased for smaller voxel sizes, as shown in Fig. 2. Both radial and tangential resolution also improved with higher values of IT×SUB where the magnitude of change from center to periphery decreased for higher IT×SUB, as shown in Fig. 3.

### B. SPECT resolution for point sources in air

**Figure 4:**
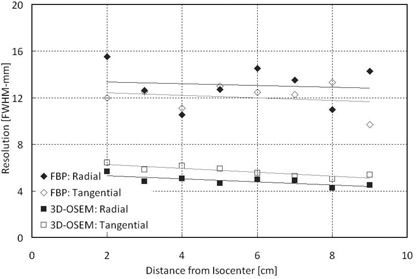
The radial and tangential resolution measured with 1.8 mm voxel size for point sources in air reconstructed with FBP and 3D OSEM with 10 iterations and 18 subsets plotted as a function of radial distance from isocenter.

To investigate the influence of iterative reconstruction on SPECT resolution, the emission data for point sources in air was reconstructed using both FBP and 3D OSEM. The radial and tangential resolution for 1.8 mm voxel size using FBP and 3D OSEM with 10 iterations and 18 subsets is plotted in Fig. 4 as a function of radial distance from isocenter. The SPECT resolution using 3D OSEM was substantially superior (FWHM was narrower by about a factor of 2) to the resolution that was achieved using FBP. This finding is not unexpected, since iterative reconstruction techniques are known to produce higher‐quality images relative to FBP,^(^ ^4^ ^)^ though the observed magnitude of improvement in ${\;}^{99m}\text{Tc}$ SPECT images was unknown.

The measured radial and tangential resolutions for point sources in air using FBP showed no variations with distance away from isocenter. A chi‐square goodness‐of‐fit test^(^ ^9^ ^)^ could not reject the null hypothesis of a constant value at the 0.05 confidence level; the p‐values were 0.48 and 0.90 for the radial and tangential resolution data, respectively. However, the same emission data when reconstructed using 3D OSEM exhibited nonstationary behavior (i.e., a linear variation in resolution with distance away from isocenter) as shown in Fig. 4.

There was no systematic difference measured between radial and tangential resolution for point sources in air using FBP, suggesting an isotropic resolution. The mean radial and tangential resolutions measured using FBP was 13.09 (11.86 to 14.32) mm and 12.05 (11.23 to 12.86) mm, respectively, where the range in the parenthesis indicates the 95% confidence intervals. However, the same emission data when reconstructed using 3D OSEM exhibited slight anisotropy; the radial resolution was measured to be slightly but consistently better (narrower FWHM but within 1 mm) than tangential resolution, as shown in Fig. 4.

### C. Dependence of SPECT resolution on the number of iterations and subsets

**Figure 5:**
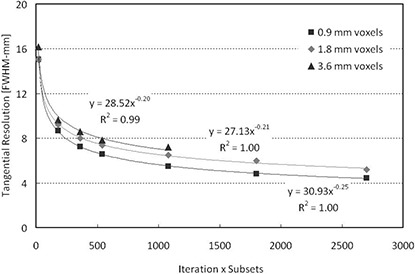
The measured tangential resolution at 6 cm from isocenter for point sources in warm background and their power‐law fits plotted against the product of iterations and subsets (IT×SUB) for different voxel sizes.

The measured radial and tangential resolution for point sources in warm background improved (narrower FWHM) in a power‐law fashion with the product of iterations and subsets (IT×SUB), for all voxel sizes and at all radial locations. Figure 5 shows the power‐law dependence of the tangential resolution measured at 6 cm from isocenter on IT×SUB for different voxel sizes.

**Figure 6:**
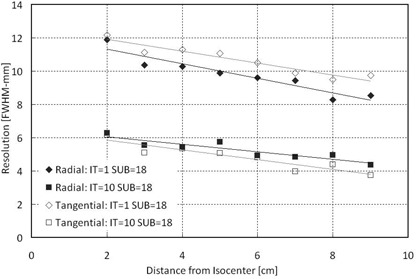
The radial and tangential resolution measured with 1.8 mm voxel size and reconstructed with 3D OSEM using two different sets of iteration (IT) and subsets (SUB) for point sources in cold background plotted as a function of radial distance from isocenter.

The convergence of spatial resolution with iterative reconstruction was found to be different between the radial and tangential components. As shown in Fig. 6, tangential resolution was worse (wider FWHM) than radial resolution at a lower number of iteration and subsets, but then improved (tangential resolution was better than radial resolution) at a higher number of iterations and subsets, suggesting that the tangential resolution converged faster than the radial resolution.

### D. Dependence of SPECT resolution on the spatial distribution of activity

**Figure 7:**
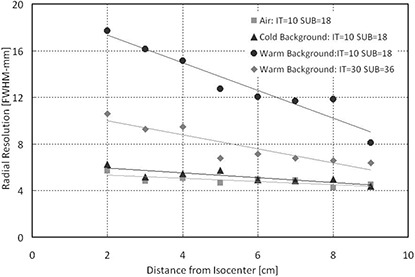
The radial resolution measured with 1.8 mm voxel size plotted as a function of radial distance from isocenter for point sources in air, cold background, and warm background reconstructed using 3D OSEM with 10 iterations and 18 subsets. The radial resolution for point sources in warm background when reconstructed with 30 iterations and 36 subsets are also shown.

The overall SPECT resolution using identical iterative reconstruction parameters was found to depend on the spatial distribution of radionuclide. Table 1 shows the measured radial and tangential resolution for point sources in air, in warm background and in cold background, when reconstructed with 3D OSEM using identical iterations and subsets. SPECT resolution with 3D OSEM was near identical for point sources in air and in cold background for a given combination of iteration and subset, as shown in Fig. 7. However, the SPECT resolution was measured to be substantially poorer (wider FWHM) when the point sources were located in a warm background. As seen in Fig. 7, a higher number of iterations and subsets were needed in SPECT reconstruction for point sources in warm background to achieve a resolution similar to that measured for point source in cold background at lower number of iterations and subsets.

**Table 1:** The measured radial (RR) and tangential (TR) resolution as a function of radial distance from isocenter for point sources in air, in warm background and in cold background, when reconstructed with 3D OSEM using 10 iterations and 18 subsets.

*Radial Distance*	*Point Sources In Air*		*Point Sources In Cold Background*		*Point Sources In Warm Background*	
*(cm)*	*RR (mm)*	*TR (mm)*	*RR (mm)*	*TR (mm)*	*RR (mm)*	*TR (mm)*
2	5.69	6.42	6.28	6.17	17.72	14.94
3	4.83	5.82	5.18	5.10	16.14	15.32
4	5.07	6.17	5.45	5.35	15.14	12.60
5	4.65	5.92	5.74	5.08	12.74	10.07
6	4.97	5.57	4.94	4.83	12.03	9.17
7	4.90	5.29	4.85	3.98	11.70	8.41
8	4.26	5.03	4.96	4.40	11.85	8.33
9	4.52	5.38	4.38	3.76	8.10	7.71

**Figure 8:**
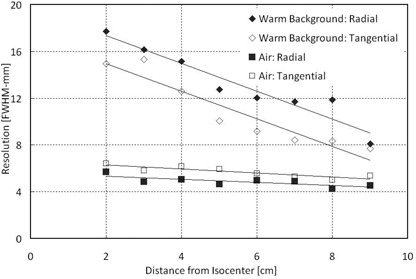
The radial and tangential resolution measured with 1.8 mm pixels and reconstructed with 3D OSEM using identical number of iterations (=10) and subsets (=18) for point sources in air and in warm background.

In addition, the sense of anisotropy in resolution was different for point sources in air (where tangential resolution was worse than the radial resolution) compared to point sources in cold or warm background (where the radial resolution was worse than the tangential resolution), as illustrated in Fig. 8. The presence of scattering and attenuating medium appears to affect differently the radial and tangential resolution in SPECT images.

## IV. DISCUSSION

**Figure 9:**
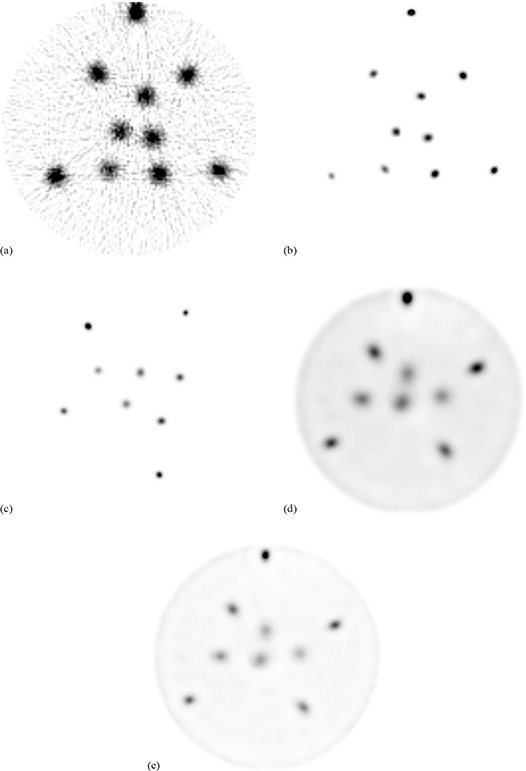
Example SPECT images with 1.8 mm voxel size to illustrate differences in SPECT spatial resolution depending on the specific activity distributions and image reconstruction technique employed. Point sources: (a) in air using FBP and (b) using 3D OSEM with 10 iterations and 18 subsets; (c) in cold background and (d) in warm background using 3D OSEM with 10 iterations and 18 subsets; (e) in warm background using 3D OSEM with 30 iterations and 18 subsets.

Figure 9 shows example SPECT images through the central slice of the point sources for the three experiments using different reconstruction parameters. The set of images in Fig. 9 are meant to illustrate the large differences in final SPECT spatial resolution depending on the specific activity distributions and image reconstruction technique employed. Images of the point sources in air are shown reconstructed using FBP in Fig. 9(a), and using 3D OSEM with 10 iterations and 18 subsets in Fig. 9(b) that clearly demonstrates the improved resolution realized with iterative reconstruction, which is consistent with the results plotted in Fig. 4. The image of point sources in cold background using 3D OSEM iterative reconstruction with 10 iterations and 18 subsets is shown in Fig. 9(c). Note the similarity in SPECT resolution for point sources in air and in cold background with 3D OSEM for a given combination of iteration and subsets; this is consistent with the results plotted in Fig. 7. The image of point sources in warm and cold background using identical iterative reconstruction schema of 3D OSEM with 10 iterations and 18 subsets yielding vastly different resolution values are shown in Figs. 9(c) and 9(d), respectively. The resulting image when the data in Fig. 9(d) (point sources in warm background) are reconstructed with a higher number of iterations and subsets, 30 iterations, and 18 subsets, is shown in Fig. 9(e). The images shown in Figs. 9(c) to 9(e) are consistent with the results shown in Fig. 7.

The 3D OSEM reconstruction for point sources in air were minimally affected by scatter and attenuation correction due to the absence of scatter media and low levels of attenuation by the glass capillary tubes. Therefore, the nonstationary resolution that was observed for point sources in air with 3D OSEM but not with FBP images (Fig. 4) was most likely due to the specific implementation of the resolution recovery algorithm in the iterative reconstruction software.^(^ ^8^ ^,^ ^10^ ^)^

As seen in Fig. 5, a convergence of the spatial resolution was not fully achieved even at the high values of iterations and subsets investigated. However, routine reconstruction at high iterations and subsets may not be practical, since it is well known that image noise also increases monotonically with IT×SUB when using iterative reconstruction.^(^ ^4^ ^,^ ^11^ ^)^ Clinical images are reconstructed using the manufacturer recommended IT×SUB values of 8 × 16 or 16 × 16 that provide a reasonable trade‐off between spatial resolution and image noise.

## V. CONCLUSIONS

SPECT spatial resolution measured using the Siemens Symbia T SPECT/CT system with Flash3D OSEM reconstruction software exhibited the following characteristics:

- Non‐stationary resolution: Both radial and tangential resolution improved with radial distance away from isocenter. The magnitude of variation decreased for smaller voxel sizes and for higher number of iterations and subsets. Stationary SPECT resolution was observed with FBP.
- Anisotropic resolution: Tangential resolution was not found to be equal to the radial resolution. The nature of the anisotropy (whether tangential resolution was better or worse than the radial resolution) depended upon the spatial distribution of the radionuclide and on the reconstruction parameters used. Isotropic SPECT resolution was observed with FBP.
- Convergence: Both radial and tangential resolution improved with higher iterations and subsets and for smaller voxel sizes. Also tangential resolution was measured to converge faster than the radial resolution with higher iterations and subsets.

In summary, SPECT spatial resolution and the resulting quantification of SPECT uptake via partial‐volume correction in clinical images depend on the nature of activity distribution within the SPECT field of view and on the specific choice of iterative reconstruction parameters (iterations, subsets, and filtration).

## ACKNOWLEDGMENTS

The author wishes to thank Bill Erwin, MS, and Osama Mawlawi, PhD, for insightful discussions.
